# Supplementary material for: A spatially resolved brain region- and cell type-specific isoform atlas of the postnatal mouse brain
Source: Nat Commun. 2021 Jan 19;12:463. doi: 10.1038/s41467-020-20343-5 (PMC7815907; doi:10.1038/s41467-020-20343-5)
Supplement: Supplementary file 1 — Supplemental Material [file 41467_2020_20343_MOESM1_ESM.pdf]

Supplemental Figure 1

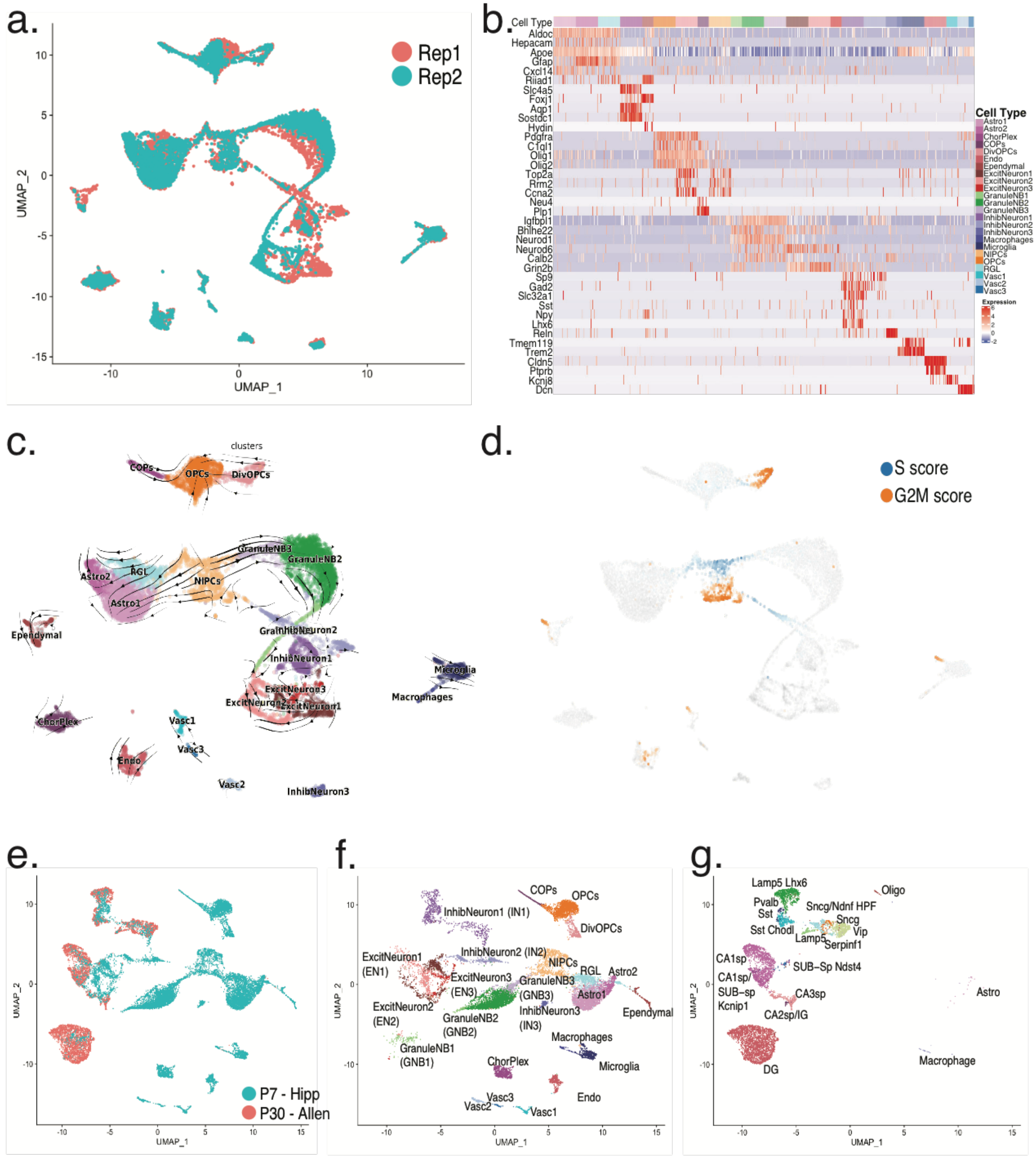

S1. Identification of hippocampal cell-types based on single-cell short read gene expression

a. UMAP of hippocampal data colored by replicate **b.** Heatmap of marker gene expression across all identified cell types. Each horizontal band represents a gene, whereas each vertical line is a randomly chosen cell. Annotated blocks of cells at the top represent cell-types **c.** RNA velocity analysis of hippocampal single-cells **d.** Cell-cycle phase identified by RNA velocity analysis **e.** Integrated UMAP from aligning above single-cell P7 hippocampus data and P30 hippocampal data from the Allen Institute colored by time-point **f.** P7 hippocampal data extracted from S1E, colored by cell-type shown in S1B **g.** P30 hippocampal data extracted from S1E, colored by cell-types identified by the Allen Institute dataset.

Supplemental Figure 2

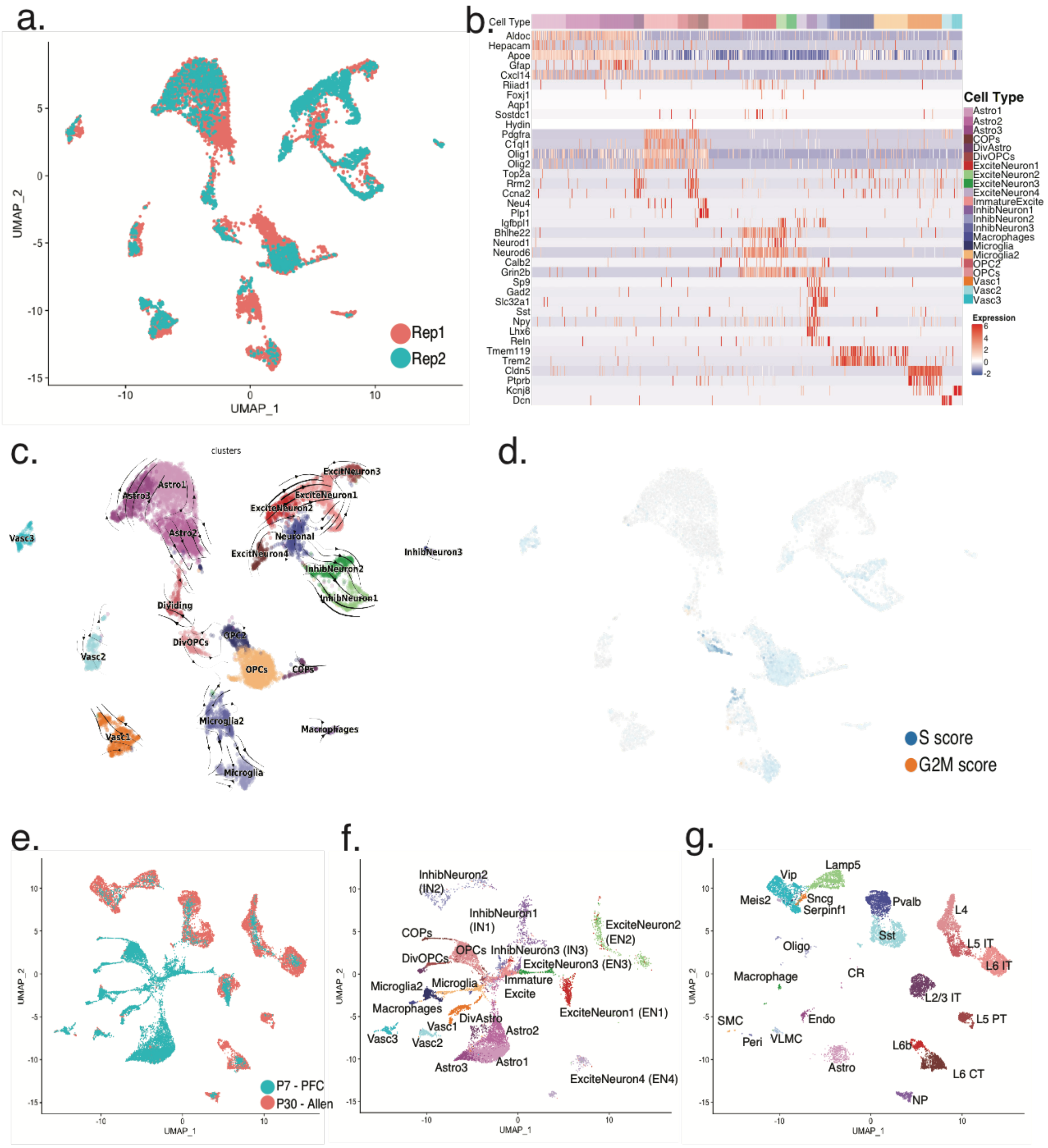

S2. Identification of prefrontal cortex cell-types based on single-cell short read gene expression
a. UMAP of hippocampal data colored by replicate **b.** Heatmap of marker gene expression across all identified cell types. Each horizontal band represents a gene, whereas each vertical line is a randomly chosen cell. Annotated blocks of cells at the top represent cell-types **c.** RNA velocity analysis of prefrontal cortex single-cells **d.** Cell-cycle phase identified by RNA velocity analysis **e.** Integrated UMAP from aligning above single-cell P7 PFC data and P30 cortex data from the Allen Institute colored by time-point. **f.** P7 PFC data extracted from S2E, colored by cell-type shown in S2B **g.** P30 cortex data extracted from S2E, colored by cell-types identified by the Allen Institute dataset.

Supplemental Figure 3

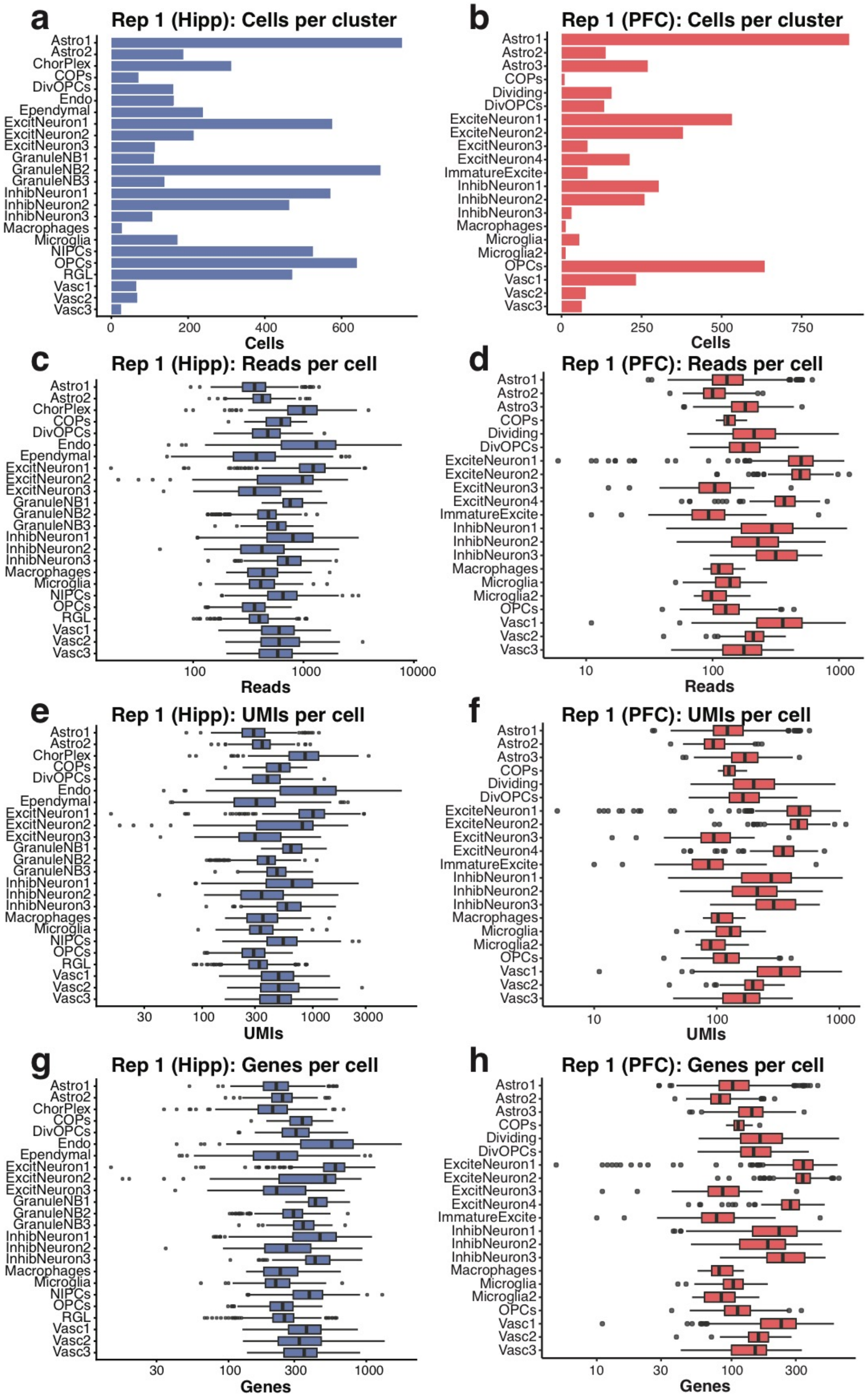

S3. Long-read statistics for Replicate 1

a. Barplot of cells per cluster identified in HIPP (n = 6892 cells) **b.** Barplot of cells per cluster identified in PFC (n = 7457 cells) **c.** Boxplot of reads per cell, grouped by cluster in HIPP **d.** Boxplot of reads per cell, grouped by cluster in PFC **e.** Boxplot of UMIs per cell, grouped by cluster in HIPP **f.** Boxplot of UMIs per cell, grouped by cluster in PFC **g.** Boxplot of genes per cell, grouped by cluster in HIPP **h.** Boxplot of genes per cell, grouped by cluster in PFC. **c-h** Data are represented as boxplots where the middle line is the median, the lower and upper hinges correspond to the first and third quartiles, the upper whisker extends from the hinge to the largest value no further than $1.5 \times$ IQR from the hinge (where IQR is the inter-quartile range) and the lower whisker extends from the hinge to the smallest value at most $1.5 \times$ IQR of the hinge, while data beyond the end of the whiskers are outlying points that are plotted individually.

Supplemental Figure 4

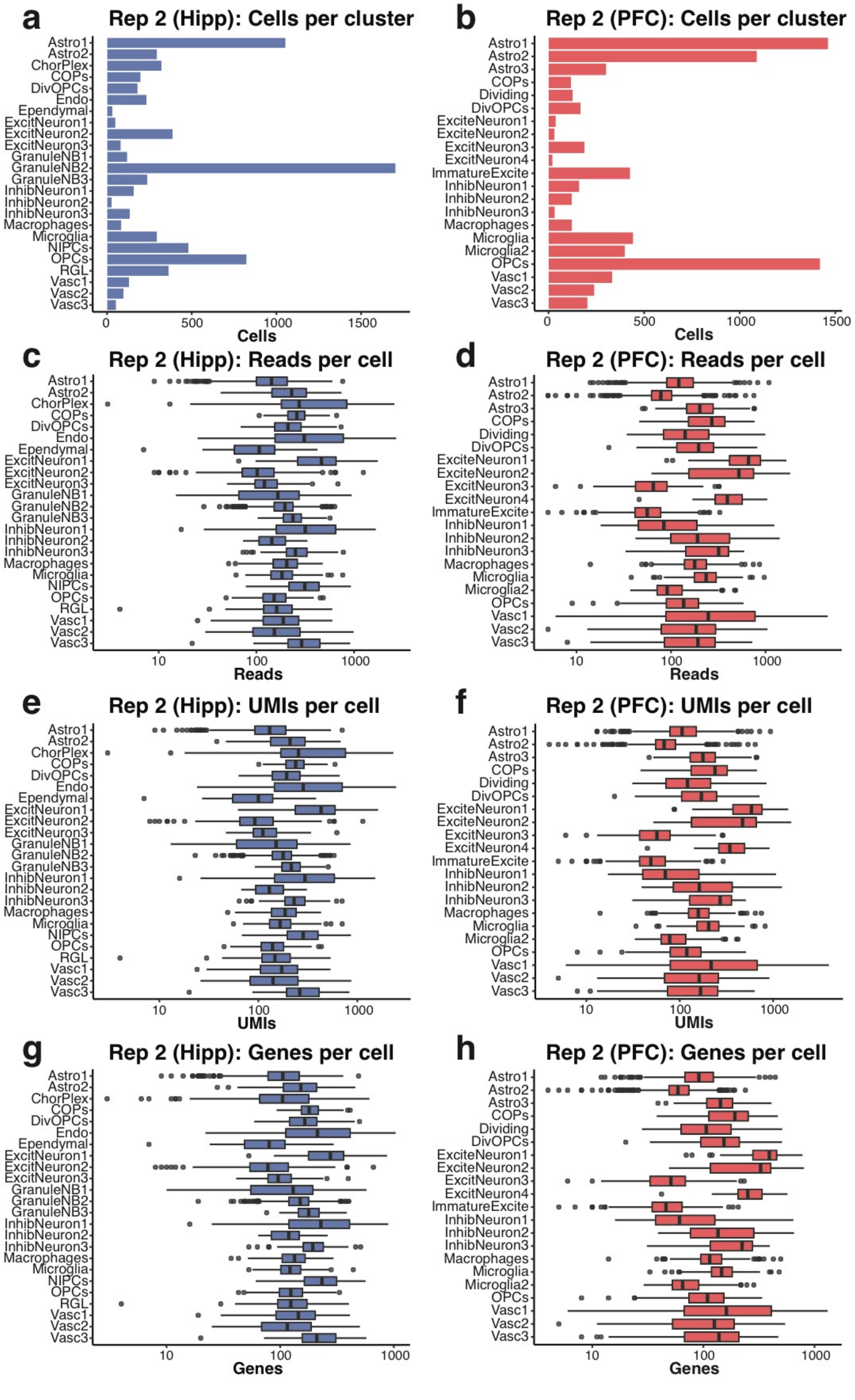

S4. Long-read statistics for Replicate 2

a. Barplot of cells per cluster identified in HIPP (n = 7541 cells) **b.** Barplot of cells per cluster identified in PFC (n = 3487 cells) **c.** Boxplot of reads per cell, grouped by cluster in HIPP **d.** Boxplot of reads per cell, grouped by cluster in PFC **e.** Boxplot of UMIs per cell, grouped by cluster in HIPP **f.** Boxplot of UMIs per cell, grouped by cluster in PFC **g.** Boxplot of genes per cell, grouped by cluster in HIPP **h.** Boxplot of genes per cell, grouped by cluster in PFC. **c-h** Data are represented as boxplots where the middle line is the median, the lower and upper hinges correspond to the first and third quartiles, the upper whisker extends from the hinge to the largest value no further than 1.5 × IQR from the hinge (where IQR is the inter-quartile range) and the lower whisker extends from the hinge to the smallest value at most 1.5 × IQR of the hinge, while data beyond the end of the whiskers are outlying points that are plotted individually.

Supplemental Figure 5

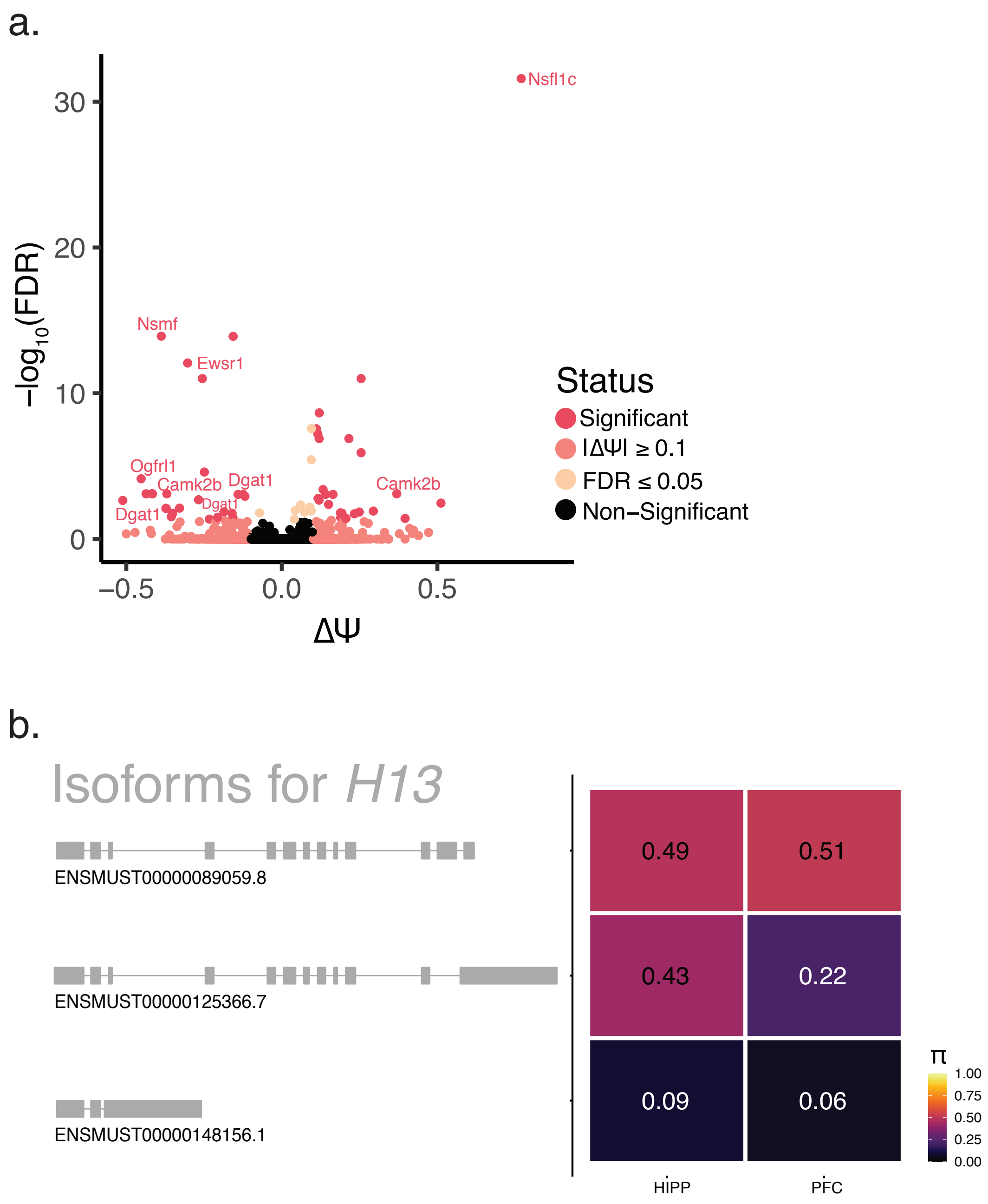

S5. Gene-wise test for DIE outperforms exon-wise test

a. Volcano plot of bulk HIPP vs. bulk PFC differential exon expression, with the effect size ($\Delta\Psi$) on the X-axis. P-values derived from a χ^2 test and corrected for multiple testing using the Benjamini-Yekutieli correction are plotted on the Y-axis. Points are colored according to the levels of significance based on the FDR and $\Delta\Psi$ value. Genes considered significant (pink) when FDR <= 0.05 and $|\Delta\Psi|$ >= 0.1 **b.** Differential isoform usage missed by exon-based tests because of harsher correction for multiple testing

Supplemental Figure 6

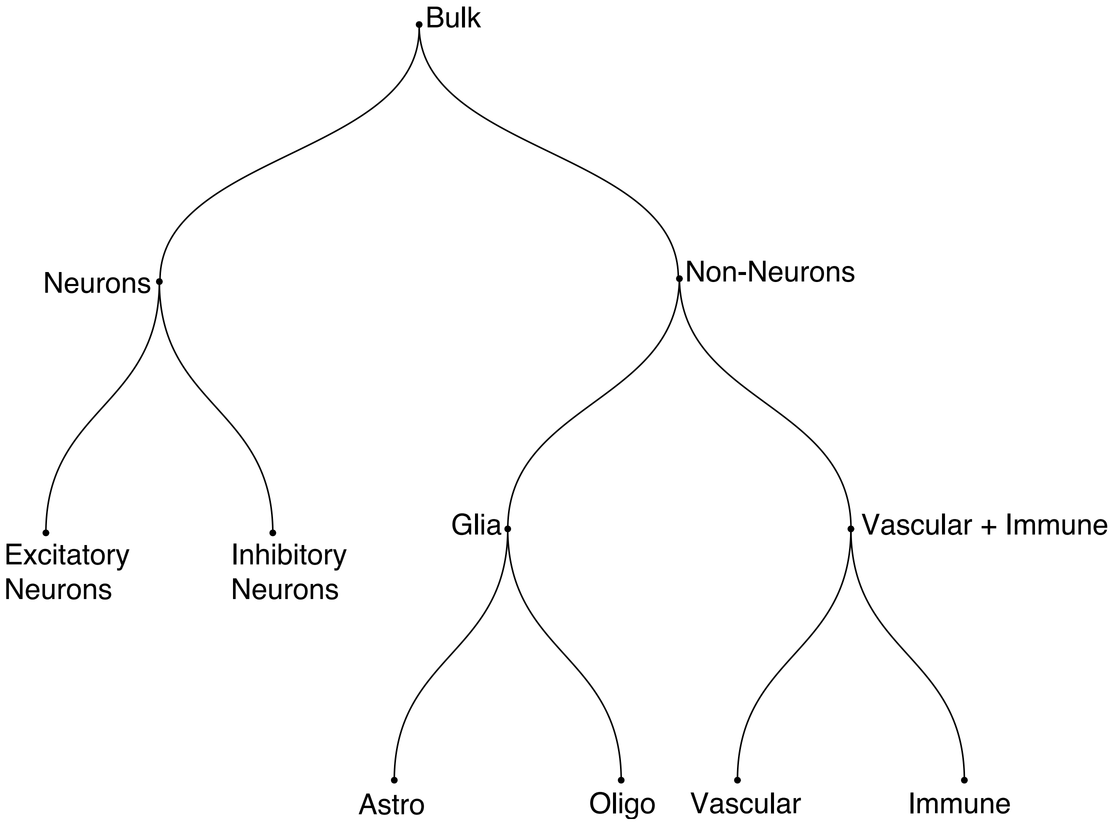

S6. Cell-type hierarchy based on gene-expression similarities
Hierarchy used for grouping cell-types together. Excitatory and Inhibitory neurons considered together in the class 'Neurons' when comparing to the 'Non-Neuron' category including glia, vascular, and immune populations.

Supplemental Figure 7

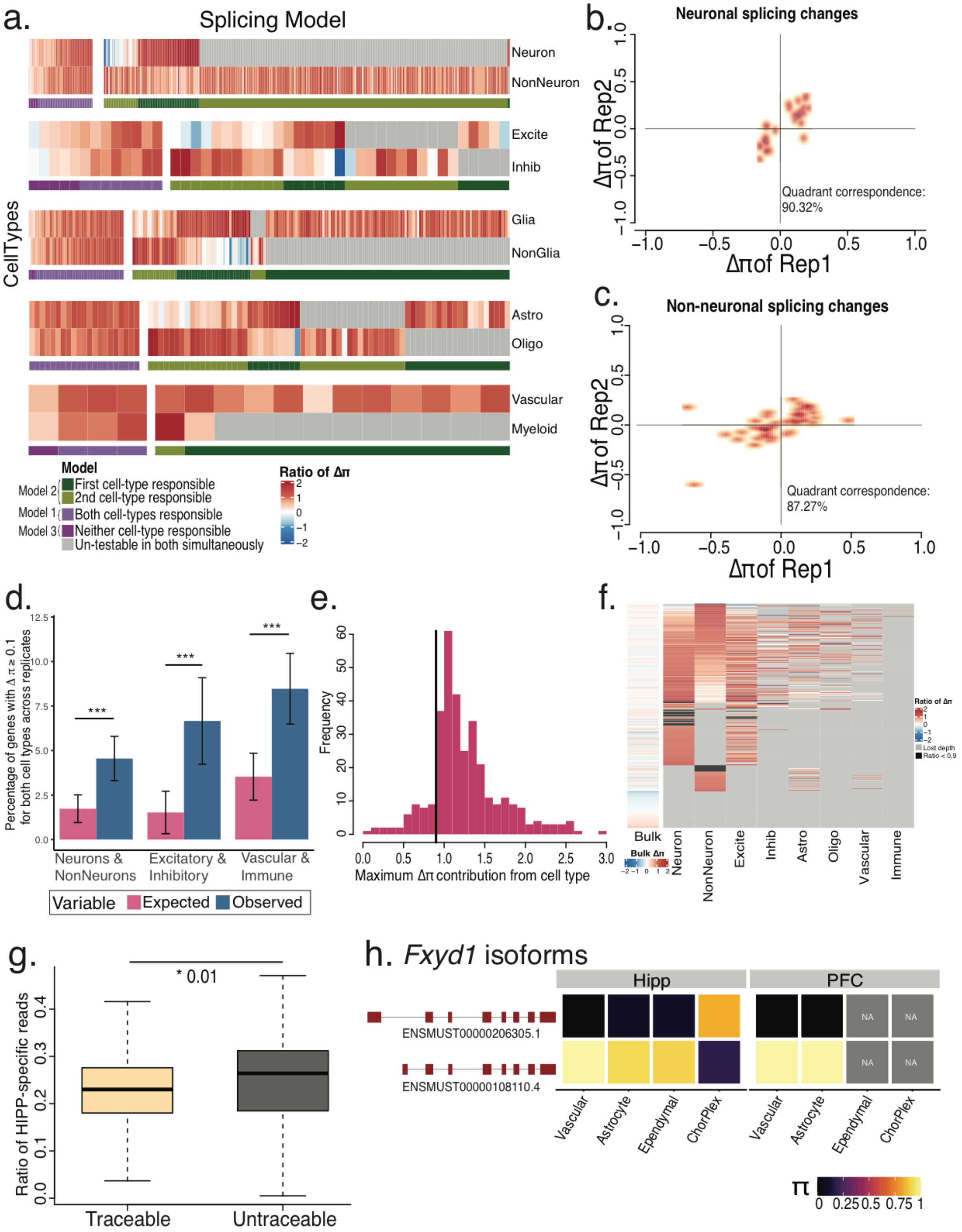

S7. One cell-type model underlies region-specific splicing differences is replicable

a. Five $gene \times celltype$ heatmaps clustered by the ratio of $\Delta\Pi$ of an individual cell-subtype to a parent cell-type. Each vertical line indicates the ratio of $\Delta\Pi$ for a single gene. Grey lines indicate lack of sufficient depth or lack of expression. Clusters of genes are colored by whether both cell-types show similar relative $\Delta\Pi$ to the parent (purple, Model I, Model III) or whether one cell-type explains most of the splicing changes (Model II) **b.** Scatter plot showing direction of neuronal region-specific splicing changes. X-axis represents $\Delta\Pi$ in neuronal cells of replicate 1. Y-axis represents $\Delta\Pi$ in neuronal cells of replicate 2 **c.** Scatter plot showing direction of non-neuronal region-specific splicing changes. X-axis represents $\Delta\Pi$ in non-neuronal cells of replicate 1. Y-axis represents $\Delta\Pi$ in non-neuronal cells of replicate 2 **d.** Barplot of expected and observed concurrent regional DIE. Concurrence is calculated as the percentage of genes with $\Delta\Pi$ of at least 0.1 in both cell-types considered, in both replicates. Expected value is calculated by assuming independence of cell-types across replicates. Pink bar indicates expected levels assuming independence, while blue bars represent observed levels. X-axis represents the three populations considered (N=1077,405,755). Error bars indicate 95% confidence interval of proportion. P-values calculated using Fisher's two-sided exact test.(***p < 10-16) **e.** Histogram of maximum differential isoform abundance contribution ($\frac{\Delta\pi_{cell-type}}{\Delta\pi_{Bulk}}$) from individual cell groups per gene **f.** Heatmap of $\frac{\Delta\pi_{cell-type}}{\Delta\pi_{Bulk}}$ for 395 region-specific DIE genes from Rep1 for six individual cell-types, and aggregated neurons and non-neurons. Each row is a gene and each column represents the indicated cell-groups. Colors indicate $\frac{\Delta\pi_{cell-type}}{\Delta\pi_{Bulk}}$ except for the bulk column, where colors represent $\Delta\Pi$. Grey indicates untestable in a cell type due to low read depth, while black indicates $\frac{\Delta\pi_{cell-type}}{\Delta\pi_{Bulk}} \leq 0.9$ **g.** Boxplots of ratio of reads originating from hippocampus specific cell-types split by genes where DIE can (N=290) or cannot be traced (N=105) to individual cell types (Rep1). P-value was calculated using two-sided Wilcoxon rank sum test. Data are represented as boxplots where the middle line is the median, the lower and upper hinges correspond to the first and third quartiles, the upper whisker extends from the hinge to the largest value no further than 1.5 × IQR from the hinge (where IQR is the inter-quartile range) and the lower whisker extends from the hinge to the smallest value at most 1.5 × IQR of the hinge. Please see function geom_boxplot in R (ggplot2) **h.** Isoform expression of two major isoforms of *Fxyd1*. Rows indicate isoforms whereas columns indicate cell type split by brain region, colored by Π value

Supplemental Figure 8

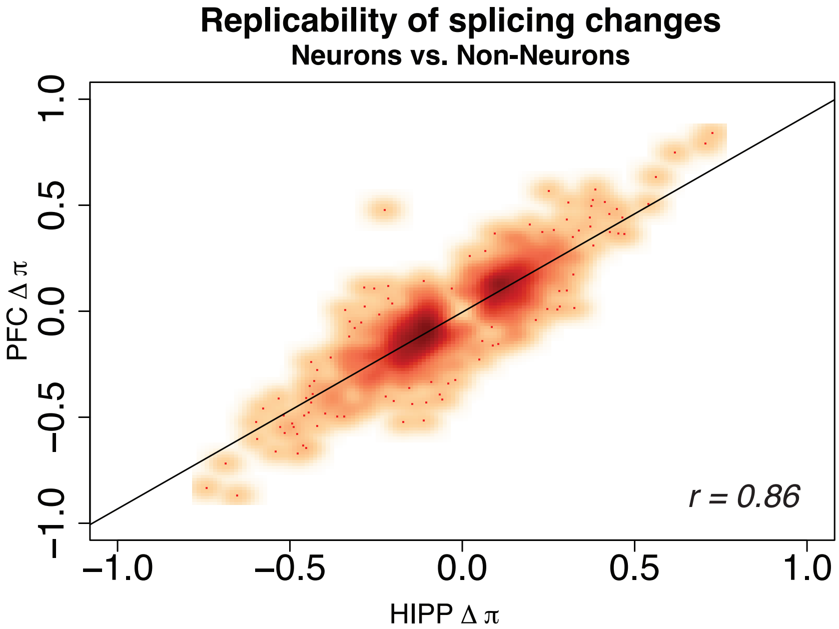

S8. Regional replicability of cell-type specific splicing changes

Scatter plot showing the direction of splicing changes between HIPP and PFC for neurons vs. non-neurons. X-axis represents $\Delta\Pi$ of neurons vs non-neurons in hippocampal cells. Y-axis represents $\Delta\Pi$ of neurons vs non-neurons in prefrontal cortex cells

Supplemental Figure 9

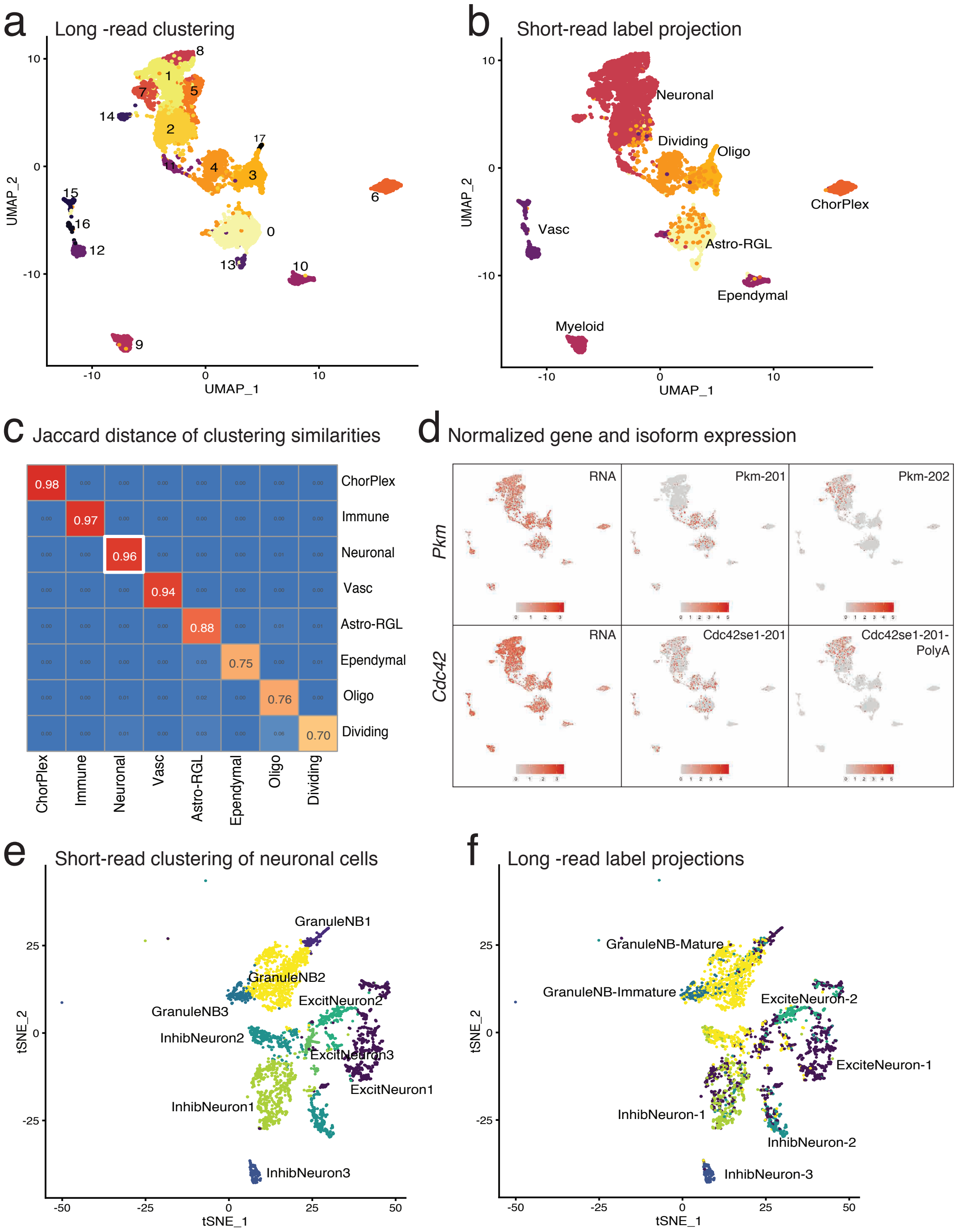

S9. Clustering on single-cell isoform expression

a. UMAP of clustering based on full-length, hippocampal single-cell transcripts where each point is a cell and color corresponds to various clusters **b.** UMAP from S9A with single-cells colored by cell-types identified by short-read gene expression from Fig1 **c.** Heatmap of jaccard similarity between cell-types identified by clustering on short reads and cell-types identified by clustering on full-length transcripts **d.** Projection of normalized gene and transcript expression for Pkm and Cdc42 on UMAP obtained from clustering on full-length transcripts **e.** Sub-setting neuronal cell-types identified from short-read expression. Clustering based on gene expression similarities between cells, and colored by short-read cell types **f.** Sub-setting neuronal cell-types identified from short-read expression. Clustering based on isoform expression similarities between cells, and colored by cell-types obtained from long-read clustering.

Supplemental Figure 10

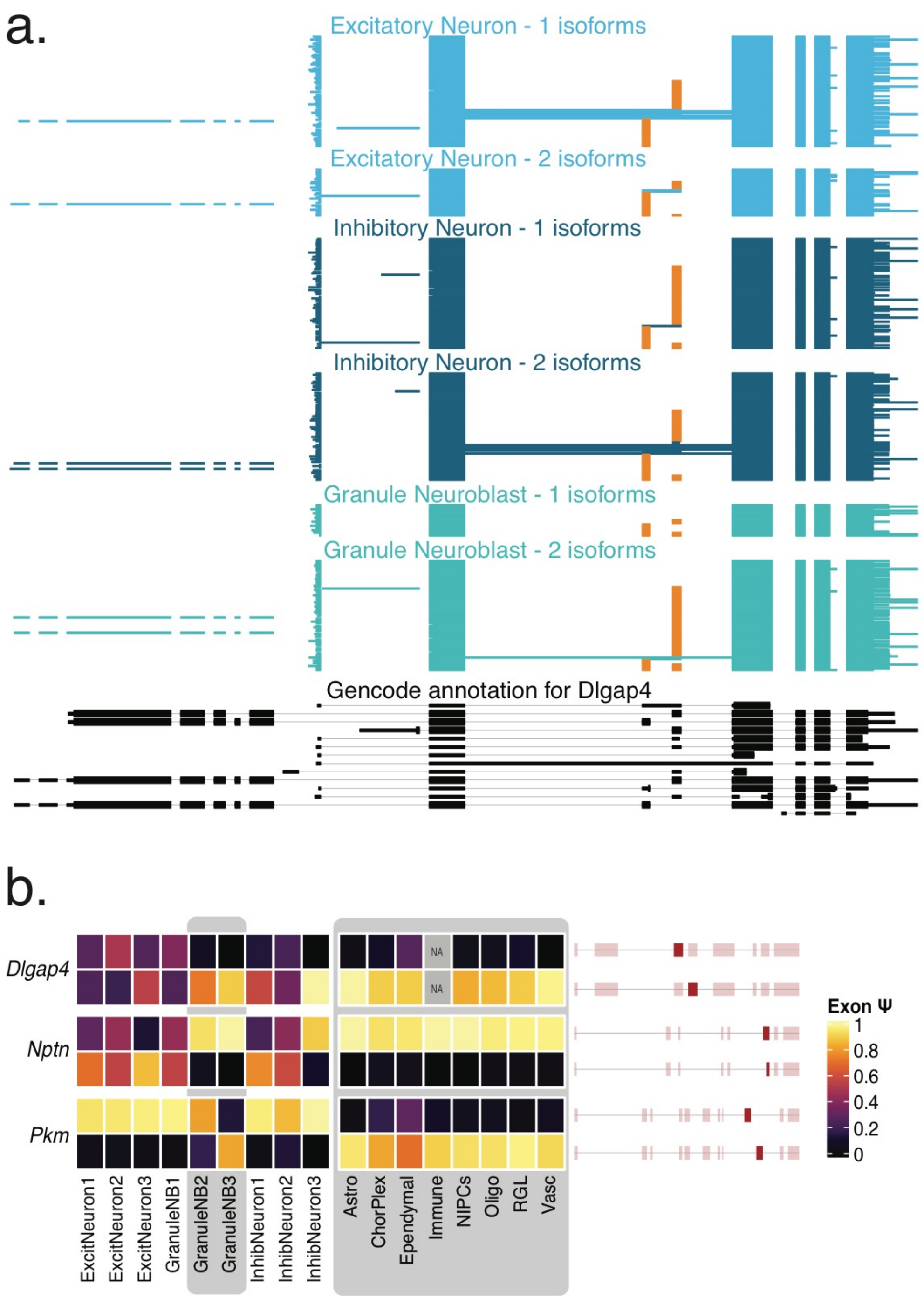

S10. Cell-type specificity of neurodevelopmentally regulated genes

a. Isoform expression for Dlgap4 gene. Each horizontal line in the plot represents a single transcript colored according to the cell-type it is represented in. Orange exons represent alternative inclusion

b. Heatmap of Ψ values for the indicated exons of *Dlgap4, Nptn*, and *Pkm*. Rows indicate exons colored by Ψ values whereas columns indicate the hippocampal cell types they are expressed in. Grey rounded boxes show non-neuronal and immature neuronal cell-types

Supplemental Figure 11

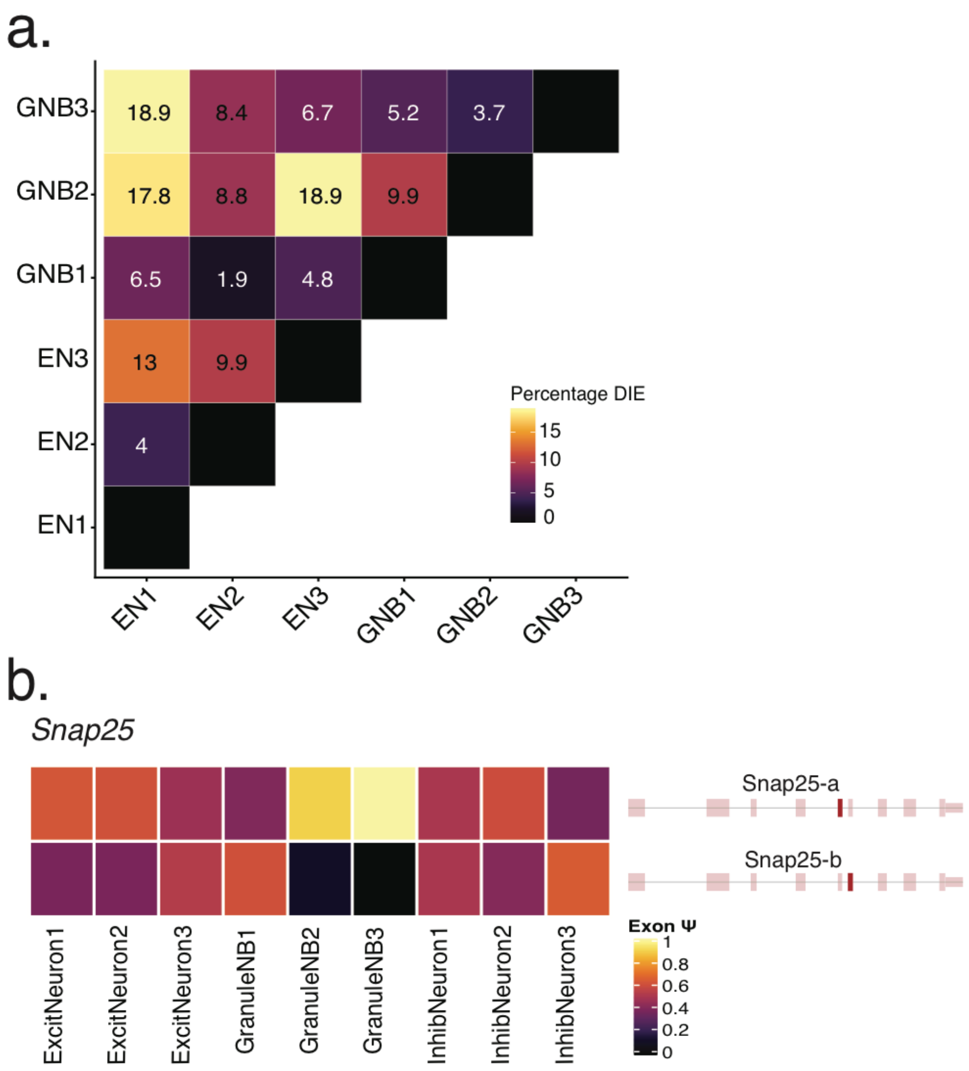

S11. Cell-type specificity of neurodevelopmentally regulated genes

a. Triangular heatmap showing percentage of DIE genes between all subtypes of GranuleNB (GNB1, GNB2, GNB3) and all subtypes of excitatory neurons (EN1, EN2, EN3) **b.** Heatmap of Ψ values for the indicated exons of *Snap25*. Rows indicate exons colored by Ψ values whereas columns indicate the neuronal cell types they are expressed in.

Supplemental Figure 12

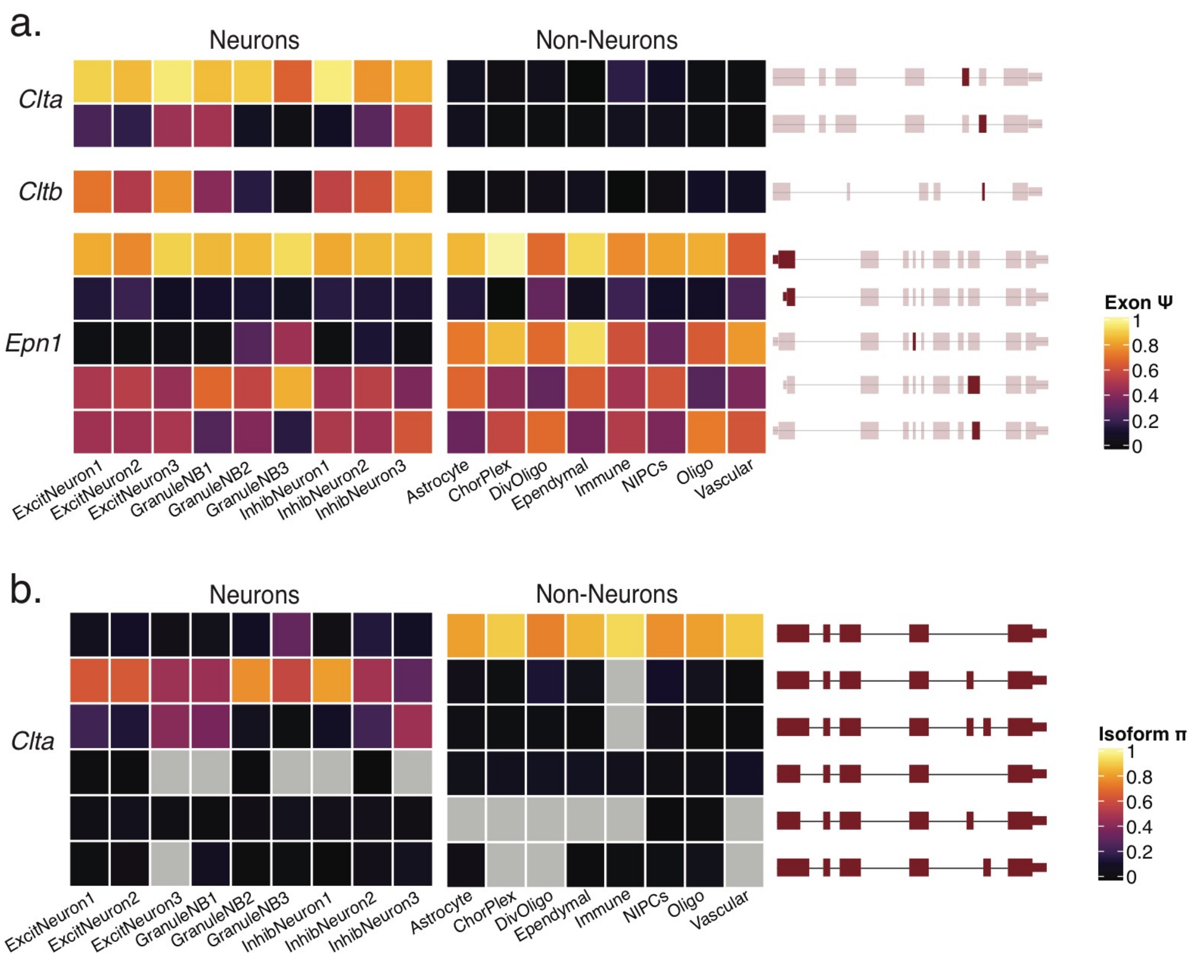

S12. Cell-type specificity of genes involved in vesicle endocytosis

a. Heatmap of Ψ values for the indicated exons of *Clta, Cltb,* and *Epn*. Rows indicate exons colored by Ψ values whereas columns indicate the hippocampal cell types they are expressed in **b.** Isoform distribution for *Clta*

Supplemental Figure 13

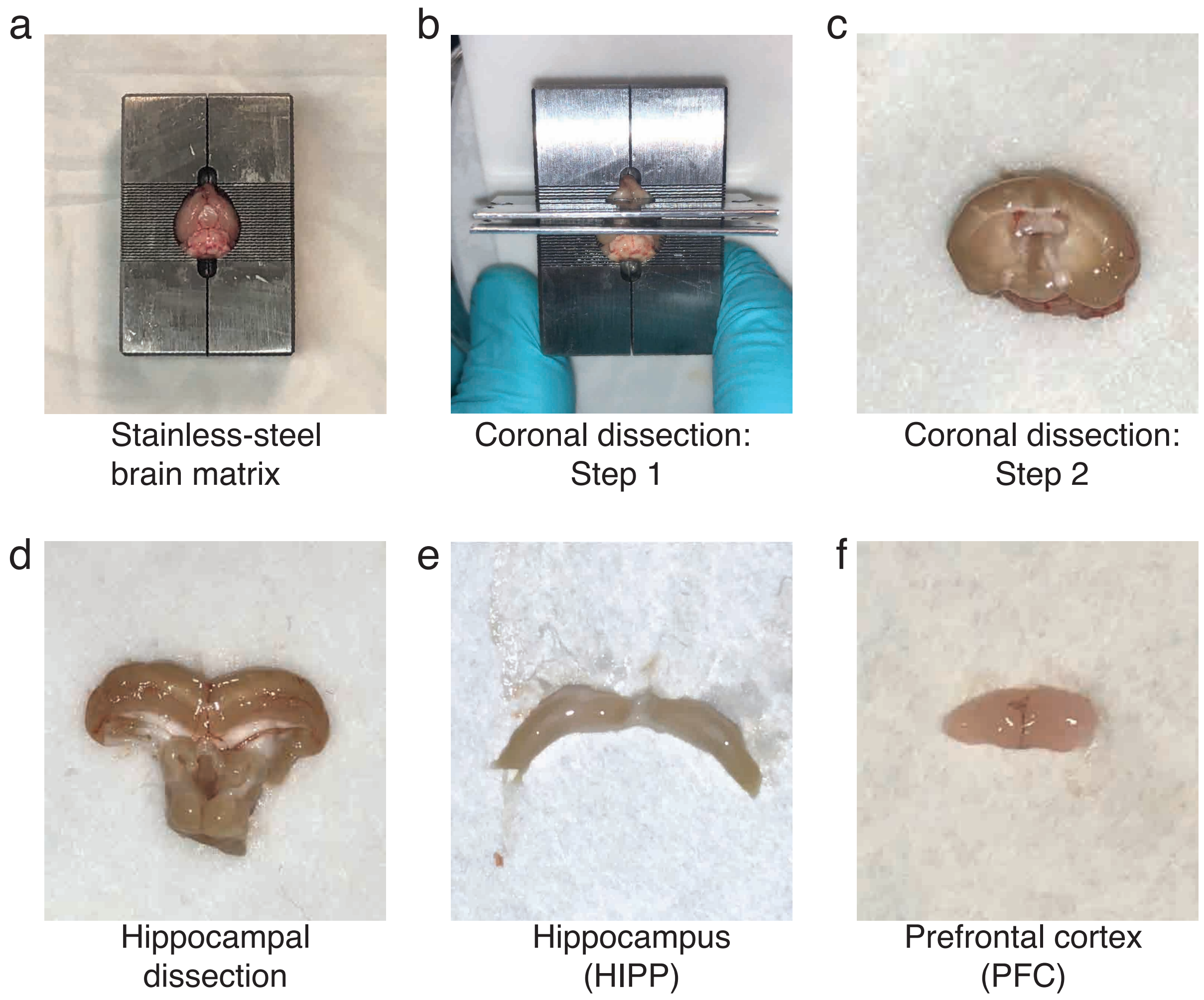

S13. Steps involved in dissecting the HIPP and PFC

a. Mouse brain placed in stainless steel brain matrix **b.** Step 1 of coronal dissection protocol **c.** Step 2 of coronal dissection protocol **d.** Dissecting out the hippocampal structure **e**. Hippocampus used in experiment **f.** PFC used in experiment

Supplemental Figure 14

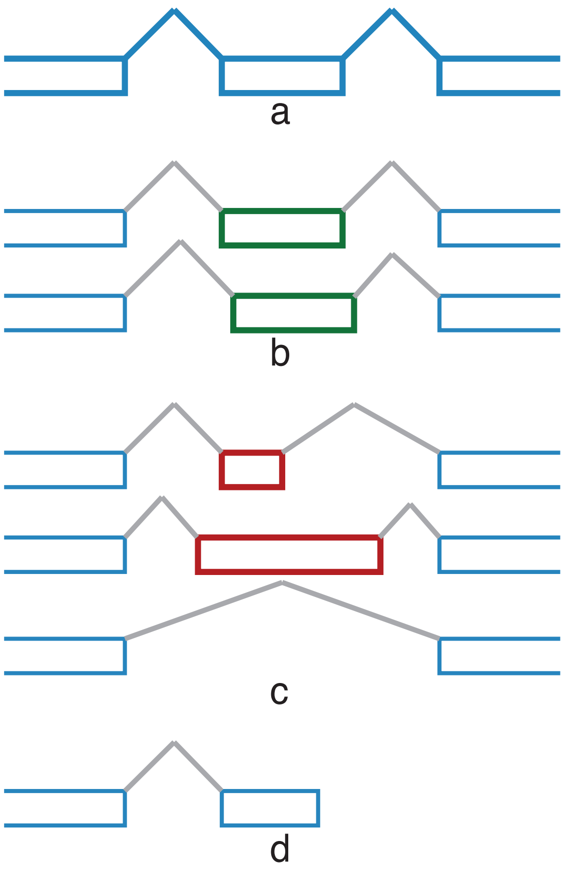

S14. Schematic showing exon counting

a. Canonical structure of isoform showing annotated internal exon **b.** 3nt difference in splice-site allowed **c.** More than 3nt difference in splice-sites not allowed **d.** Partially covered internal exons not allowed

Supplemental Table 1

Replicate	Region	# reads	Barcoded	Spliced	Barcoded & Spliced	Full-length
Rep1	HIPP	38297066	15935252	14005900	6698339	4565208
Rep1	PFC	10730073	3968311	4069936	1456689	1224345
Rep2	HIPP	12062107	5456753	5301119	2321631	1809583
Rep2	PFC	9508271	3866334	3943532	1662952	1301187
